# Comparing the Outcomes of Stapled Transanal Rectal Resection, Delorme Operation and Electrotherapy Methods Used for the Treatment of Obstructive Defecation Syndrome

**Published:** 2014-09

**Authors:** Neda Mirabi, Mohammad Fazlani, Reza Raisee

**Affiliations:** Department of General Surgery, Imam Khomeini Hospital, Tehran University of Medical Sciences, Tehran, Iran

**Keywords:** Obstructive, Resection, Iran

## Abstract

**Background: **Pathophysiology and treatment of obstructive defecation syndrome (ODS) remains to be defined clearly. Rectal hidden intussusceptions and voluminous hemorrhoids may be the cause. Where conservative treatment is not effective, ODS can be treated by STARR or Delorme operation. In some patients treatment of advance hemorrhoidal disease may resolve the syndrome.

**Methods: **81 females out of 183 ODS patients were selected for the treatment by Delorme, STARR or 30 mAmp electrotherapy.

**Results:** The number of patients treated by STARR, Delorme and Electrotherapy were 34, 31 and 16, with mean postoperative pain ranking of 2.5, 3.7 and 1.5 and mean hospital stay of 2.3, and 3.2 and 1 day respectively. Mean ODS score, preoperatively compared with one-year post operation, improved from 14.5 to 5.1 (P=0.005) in STARR, 13.8 to 4.3 (P=0.006) in Delorme and 14.2 to 12.8 (P=0.725*)* in electrotherapy groups. The mean severity score (SS) changed from 14.2, 15.18 and 13.90 preoperatively to 3.8, 4.12 and 11.34 postoperatively in all groups respectively. The mean resting pressures decreased from 82 to 65 in STARR (P=0.006*)*, from 87 to 63 in Delorme (P=0.005*)* and from 79 to 74 mmHg (P=0.797*)* in electrotherapy groups. Postoperative defecography showed significant reduction in the intussusception parameter in STARR and Delorme (82.4% and 88% respectively; P<0.0001), but unchanged in electrotherapy group.

**Conclusion: **STARR and Delorme are effective modalities for the treatment of patients with ODS, while STARR is simpler, less invasive and less painful. Although, electrotherapy eradicates the voluminous hemorrhoids but is ineffective in the treatment of ODS.

## Introduction

Many patients with constipation suffer from obstructive defecation syndrome.^1^^,^^2^ These patients may complain about incomplete evacuation, frequent defecation, sense of obstruction of canal, tenesmus and bleeding.^1^^,^^2^

Distal hidden rectal intussusception and rectocele are the most known organic causes of obstructive defecation syndrome (ODS).^1^^-^^3^ Advance hemorrhoidal disease may also be the cause of ODS in some patients.^4^^,^^5^ If conservative treatment remedies are exhausted, these causes can be corrected surgically.^3^ Trans-rectal mucosal resection or fixing it to underneath tissue and eradicating the hemorrhoid tags can be modalities of choice.^1^^,^^3^^,^^6^ For hidden prolapse, the surgical treatment modalities may be Stapled Transanal Rectal Resection (STARR) procedure which is introduced as a new treatment approach or Delorme operation.^7^^-^^10^ For eradication of hemorrhoids, traditional hemorrhoidectomy or new methods like DG HALL or electrotherapy could be used. Using the latter two recent methods, mucosa is not removed and not sloughed but adhered to underneath tissue. Therefore, using one of the above modalities the ODS syndrome may be eliminated.^6^^,^^7^

STARR is advocated as a new technology for the treatment of obstructive defecation syndrome by excision of parts of intussusceptum. Delorme operation is a known method for the treatment of rectal prolapsed in which mucosa of prolapsing segment is removed and rectal muscle wall plicated.^3^^,^^8^^,^^11^^,^^12^ This results the elimination of blockage. Electrotherapy of hemorrhoids is used for the treatment of hemorrhoids^13^^,^^14^ and may be effective in ODS patients by fixing the loose upper canal mucosa to underlining tissue and eradication of voluminous hemorrhoidal tags which prevent normally easy defecation.^4^^,^^5^

In STARR method, an expensive instrument is used and its efficacy seems to be somewhere between 85% and 93%, but Delorme operation with success rate of 74% to 94%^15^ is a more difficult method than STARR. Electrotherapy method is easy to use and inexpensive.^13^^,^^14^

In this retrospective study the clinical outcome (resolving signs and symptoms of obstructive defecation syndrome) of different modalities, stapled transanal rectal resection, Delorme operation and electrotherapy of hemorrhoids are assessed. These modalities are currently used as surgical treatment of ODS at Imam Khomeini Hospital. This investigation aims at exploring the effectiveness of each method with respect to the pathology. 

## Patients and Methods

In this retrospective study, the clinical charts of 183 patients, all female, with signs and symptoms of ODS referred to the colorectal clinic of Imam Khomeini Hospital during September 2007 to September 2011 were reviewed. Patients were evaluated using standardized clinical and paraclinical investigations such as anoscopy, colonoscopy, defecography, colon transit time, endoanal sonography and anal manometry. 81 patients with diagnosis of hidden rectal prolapse or prolapsing circumferential hemorrhoids were selected for surgical treatment by Delorme operation, stapled transanal rectal resection (STAAR) or 30 mAmp electrotherapy^13^^,^^14^ according to the preference of consultant surgeon and clinical findings.

Patient’s problems and functional outcome were measured preoperatively and at least once postoperatively during the first year by Wexner score,^16^ symptom severity score (table 1) and obstructed defecation syndrome score (table 2).

**Table 1:** Symptom severity score*

	**None of the time**	**A little of the time**	**Some of the time**	**Most of the time**	**All of the time**
Need for laxatives/enemas	0	1	2	3	4
Unsuccessful attempts to open bowels	0	1	2	3	4
Low frequency of bowel movements	0	1	2	3	4
Increased time or straining to open bowels	0	1	2	3	4
Pain on opening bowels	0	1	2	3	4
Incomplete bowel opening	0	1	2	3	4
Bleeding on bowel opening	0	1	2	3	4
Incontinence/soiling	0	1	2	3	4
Difficulty to withstand urge to open bowels	0	1	2	3	4

*The maximum score is 36

**Table 2:** Obstructed defecation score

Frequency of defecation	
1–2 defecations every 1–2 days	0
2 defecations/week or 3 defecations or attempts/day	1
1 defecation/week or 4 defecations or attempts/day	2
1 defecation/week or 4 defecations or attempts/day	3
Intensity of straining	
No or light	0
Moderate	1
Intense	2
Duration of straining	
Short	1
Prolonged or many times	2
Incomplete evacuation	
Never	0
1 time/week	1
2 times/week	2
3 times/week	3
Rectoperineal discomfort	
Never	0
1 time/week	1
2 times/week	2
3 times/week	3
Reduction of activities	
None	0
25%	2
25–50%	4
50%	6
Laxatives	
Never	0
25% of defecations	1
25–50% of defecations	3
50% of defecations	5
Always	7
Enemas	
Never	0
25% of defecations	1
25–50% of defecations	3
50% of defecations	5
Always	7

Dynamic defecography was carried out preoperatively and postoperatively to assess anatomical abnormality in each patient.

*Statistical Analysis*

Statistical analysis was performed using paired *t* test for continuous variables with checking normality of data set, and Wilcoxon’s signed-rank test for qualitative variables and McNemar‘s test for percentages. The total scores of ODS and SS were expressed as mean values with 95% confidence intervals (CI). A P<0.05 was considered statistically significant.

## Results

The number of patients for each modality were; 34 for STARR operation (age 36 to 63 with normal vaginal delivery of 2 to 5), 31 for Delorme operation (age 34 to 61 with normal vaginal delivery of 2 to 5) and 16 for electrotherapy (age 37 to 54 with normal vaginal delivery of 1 to 4).

Mean operating time was 43 minutes for STARR, 78 minutes for Delorme operation and 18 minutes for electrotherapy. Postoperative hemorrhage occurred at the staple line of STARR group in 7 patients (16.2%) and at the suturing line of Delorme group in 2 patients (6.5%). One patient in STARR group required surgical intervention in the first 24 hours due to persistent bleeding. Mean postoperative pain score on a scale from 0 to 10 was 2.5 in STARR, 3.7 in Delorme and 1.5 in electrotherapy group. Mean hospital stay was 2.3 days for STARR and 3.2 days for Delorme and 1 day for electrotherapy group. 

Data were collected from standardized questionnaires for the clinical staging of ODS for subsequent surgery and for the evaluation of therapeutic results.^2^ The validated constipation scoring system (CSS), obstructed defecation syndrome (ODS) score system (table 1) and symptom severity (SS) score (table 2) were used for clinical assessment. Defecography was used as paraclinical assessment in this study.

The mean ODS score, before surgery compared with the one year follow-up, improved from 14.5 to 5.1 (P=0.005) in STARR, from 13.8 to 4.3 (P=0.006) in Delorme and from 14.2 to 12.8 (P=0.725) in electrotherapy groups (table 3).The mean SS score in STARR, Delorme and Electrotherapy groups was 14.2, 15.18 and 13.90 preoperatively compared with post-operative which was 3.8, 4.12 and 11.34 respectively (table 3).

**Table 3:** The scoring results in patients before and after operation

	**ODS** **Preoperation-postoperation**		**SSS** **Preoperation-postoperation**		**P value**
STARR	14.5	5.1	14.2	3.8	0.001
Delorme	13.8	4.3	15.18	4.12	0.001
Electrotherapy	14.2	12.8	13.90	11.34	0.725

ODS: Obstructed defecation score; SSS: Symptom severity score; STARR: Stapled transanal rectum

Postoperative defecography showed significant reduction in the intussusception parameter in STARR and Delorme groups (82.4% and 88% respectively; P<0.001), but insignificant change in electrotherapy group. The mean anal canal manometric resting pressures revealed significant reduction in STARR and Delorme groups but not in electrotherapy group (table 4).

**Table 4:** Mean manometric resting pressures (mm Hg)

	**Preoperation**	**6 months postoperation**	**P value**
STARR group	82	65	0.006
Delorme group	87	63	0.005
Electrotherapy group	79	74	0.797

STARR: stapled transanal rectum resection

In a mean follow-up of 18 months (range 12-46), ODS persisted or recurred clinically and/or radiologically in 12 patients (35%) in STARR, 8 patients (25%) in Delorme and 13 patients (81%) in electrotherapy groups.

Five patients (14.7%) in STARR group reported urge incontinence while they had preoperative normal continence. 

## Discussion

Obstructed defecation syndrome (ODS) is characterized by a spectrum of symptoms including prolonged straining during defecation, frequent defecation, sense of incomplete evacuation, use of external aid for defecation, tenesmus, anal pain and bleeding.

It has been estimated that approximately 15-20% of adult female population suffers from this syndrome.^10^^,^^17^^,^^18^ The etiology of ODS is multifactorial; an interaction of anatomical and functional factors that influence the recto-anal evacuatory mechanism.^2^^,^^19^ The most common pathophysiologic alterations associated with ODS are rectal intussusception and rectocele.^2^^,^^6^

Internal rectal prolapse (intussusception) is the result of chronic strain for defecation, due to chronic constipation or pelvic floor dysfunction, which develops lengthening of the attachments of rectum to sacrum leading to descending perineum syndrome.^2^^,^^20^ Intussusception is the result of increased mobility of the rectum and causes outlet obstruction, since the upper rectum moves away from the sacrum and pushes into the more distal rectum.^1^ The advance circumferential hemorrhoidal tags may be the other cause of ODS^4^^,^^5^ and should be considered in the treatment of this disease.

Different modalities such as conservative therapy, biofeedback and surgery are used to treat ODS.^6^ Although different surgical modalities have been described in the treatment of ODS, but many of these procedures are not suitable in patients with hidden rectal intussusception.^21^ STARR has been demonstrated as a relatively noninvasive surgical technique for ODS caused by rectal intussusception.^3^^,^^6^^,^^11^ However, some authors have the idea that details on the anatomical changes produced by STARR and its correlation with success or failure is poorly understood.^8^^-^^10^^,^^20^ But this novel procedure aims to resect internal prolapse, correct rectal volume, restore anatomy and improve function.^1^^,^^11^^,^^12^

Delorme operation is used in the past for the treatment of hidden rectal prolapsed^22^ and stapled transanal rectal resection was introduced as a new technology for the management of obstructive defecation syndrome.^10^^,^^21^ STARR is becoming increasingly popular for the treatment of this syndrome.^1^^,^^21^

Clinical studies have demonstrated the effectiveness and safety of this modality of treatment, but there is still no clear evidence that STARR procedure provides symptom resolution in ODS patients.^4^^,^^8^^-^^10^

Large hemorrhoidal tags may obstruct the canal and such patients have difficult defecation which may be resolved by the treatment of hemorrhoids.^8^ Electrotherapy is a modality of choice for the eradication of hemorrhoid disease, which may also attach the loose upper canal mucosa to underlining tissue by fibrosis. Electrotherapy is used in 16 -30 mAmps, however it is indicated that using 30 mAmp is more effective in a short time of application.^13^^,^^14^

In this study the effectiveness and safety of stapled transanal rectum resection (STARR) is evaluated. STARR is compared with Delorme operation as a previously used method in the treatment of obstructed defecation syndrome (ODS). It is used in conjunction with electrotherapy as a simple, minimally invasive and inexpensive instrumental method in the treatment of this disease.

Compared with the preoperative results in the current three groups of patients, obvious reduction in the Longo’s ODS and SS scores were exhibited in STARR and Delorme groups during the 12 months after surgery (table 3). Reduction in anal manometry resting pressure is also significant in STARR and Delorme methods but not in electrotherapy method (table 4).Variations demonstrates the efficacy of STARR and Delorme in relieving symptoms of obstructed defecation but not in electrotherapy. However, a few studies have reported the limited effect of STARR in treatment of ODS.^23^^-^^25^ However, in this study the effectiveness and safety of STARR is significant. Similar results were observed in Delorme group, but Delorme operation is more time consuming, invasive and difficult with more postoperative pain. Moreover, STARR results in shorter mean hospital stay. The results in patients in electrotherapy group were insignificant. Although, the hemorrhoidal tags disappeared; but patients poorly responded to this method. Post operation defecography also revealed significant reduction in the size of intususception in patients compared with pre operation in STARR and Delorme groups.

## Conclusion

STARR and Delorme procedures can be performed safely without major morbidity. These procedures are effective treatments for patients with obstructed defecation associated with symptomatic rectal intussusception. However, STARR procedure is simpler, less invasive and less painful with shorter hospital stay. Electrotherapy method eradicate the hemorrhoidal tags but is ineffective in the treatment of ODS.
